# The Role of the Gut Microbiota in Health, Diet, and Disease with a Focus on Obesity

**DOI:** 10.3390/foods14030492

**Published:** 2025-02-04

**Authors:** Zheng Feei Ma, Yeong Yeh Lee

**Affiliations:** 1 Centre for Public Health, School of Health and Social Wellbeing, College of Health, Science and Society, University of the West of England, Bristol BS16 1QY, UK; 2 School of Medical Sciences, University Sains Malaysia, Kota Bharu 15200, Malaysia

**Keywords:** gut microbiota, dysbiosis, gastrointestinal tract, metabolism, obesity

## Abstract

The gut microbiota has been increasingly recognised as a critical determinant of human health, influencing a wide range of physiological processes. A healthy gut microbiota is essential for maintaining metabolic, immune, and gastrointestinal homeostasis, contributing to overall well-being. Alterations in its composition and functionality, often referred to as microbial dysbiosis, are strongly associated with the development of gut-related and systemic diseases. The gut microbiota synthesises several components and interacts with epithelial cell receptors, influencing processes that extend beyond nutritional status to the pathogenesis of diseases such as obesity, which extend beyond their known contribution to nutritional status. Therefore, this state-of-the-art review synthesises findings from recent studies on the composition, functions, and influencing factors of the gut microbiota, with a focus on its role in obesity. A systematic search of peer-reviewed literature was conducted to ensure comprehensive coverage, while expert insights are incorporated to discuss emerging research directions and future perspectives in the field.

## 1. Introduction

**Figure 1:**
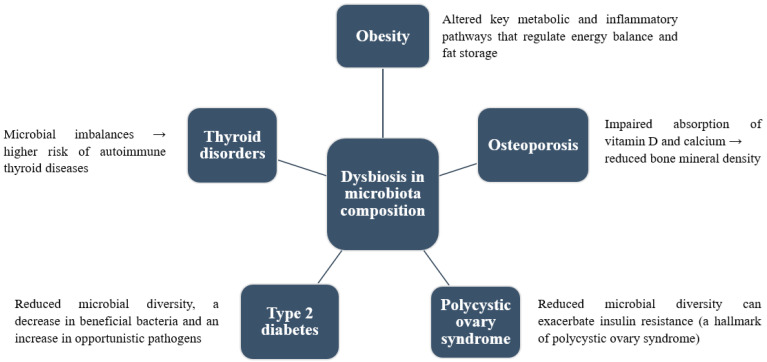
Dysbiosis in microbiota composition is associated with several diseases.

The gut microbiota, a complex and diverse ecosystem, is predominantly found in the large intestine. Gut microbiota can evolve and adapt with their hosts throughout their lifespans. In addition to within-host evolution, the gut microbiota also performs vital physiological functions for its host, including nutritional absorption and metabolism [1]. Therefore, any functional or compositional changes within gut microbiota are associated with changes in immune and metabolic functions [2]. For example, the occurrence of irritable bowel syndrome (IBS) and inflammatory bowel disease (IBD) are associated with an altered gut microbiota. In individuals with type 2 diabetes (T2D), the proportions of *Firmicutes* and *Clostridia* are significantly reduced, while the ratios of *Bacteroidetes* to *Firmicutes*, *Bacteroides-Prevotella*, and *Betaproteobacteria* are markedly increased, with these changes positively correlating with plasma glucose levels [1] (Figure 1).

Although some dominant bacterial species are usually present in most, the composition of gut microbiota can still vary greatly from individual to individual. Even the same diet consumed by two individuals can result in distinct metabolic health outcomes and personalised microbial responses. The gut microbiota changes over time and there are differences in gut microbiota composition between younger and older individuals [2]. Gut microbiota differ between individuals because of several factors, including diet. For example, diets high in carbohydrate and fibre increase the diversity and abundance of intestinal microorganisms, whereas diets low in carbohydrate lower the abundance of butyric-acid-producing bacteria including *Bifidobacterium* and *Roseburia.* An increased intake of fermented milk and prebiotics has been shown to enhance the abundance of *Bifidobacterium* within the gut microbiota [3,4]. In addition, an immature gut microbiota has been associated with malnutrition. It is suggested that permanent, rather than short-term or temporary changes in the gut microbiota composition are needed to observe their long-term effects on health outcomes [5].

Therefore, in this state-of-the-art review, we will first define the gut microbiota and outline methods for its sampling and characterisation. Next, we will discuss the composition and functions of the gut microbiota, highlighting the influence of various factors, particularly diet. Using obesity as an example, we will explore the complex relationship between the gut microbiota and health. Finally, we will explicitly address the emerging perspectives and propose directions for future research on gut microbiota. Unlike existing reviews, our work integrates recent advancements in microbiota profiling technologies, such as metagenomics and metabolomics, with personalised nutrition approaches to offer a more comprehensive understanding of individual variability in gut-microbiota responses.

### Search Methodology

To summarise the current scientific literature on gut microbiota, nutrition, and health, a comprehensive was conducted using the PubMed, Scopus, and Web of Science databases. Relevant articles published until 2024 were identified using the following keyword combinations: ‘gut microbiota’, ‘gut’, ‘dysbiosis’, ‘diet’, ‘nutrition’, ‘health’, ‘disease’, ‘diseases’, ‘non-communicable diseases’, and ‘obesity’. Publications such as editorials, commentaries, and conference abstracts lacking original data were excluded from the review.

## 2. Sampling Methods for Gut Microbiota

The ideal sampling used for collecting faecal samples should have the following characteristics: non-invasive and no or little cross contamination/bowel preparation [4]. Faecal samples collected from individuals are used as a proxy for gut microbiota. This is because they are non-invasive, and naturally and repeatedly collected (Table 1).

Compared with the collection of faecal samples, the composition of gut microbiota can also be analysed by collecting tissue samples and luminant contents through endoscopic procedures (e.g., biopsy and luminal brushing). However, endoscopic procedures are not friendly to participants and are invasive. On the other hand, there are also some new sampling technologies including the Brisbane Aseptic Biopsy Device and intelligent capsule which can be used to provide a more precise assessment of gut microbiota [4]. In addition, different sites such as faeces, intestinal fluid, and mucosal biopsy should be used to collect the samples. This is because the host immune activity and physiological functions are different in the small and large intestines, and this can influence gut microbiota composition. The small intestine is dominated by *Enterobacteriaceae* and *Lactobacillaceae*, while the large intestine predominately contains *Rikenellaceae*, *Bacteroidaceae*, *Lachnospiraceae*, *Prevotellaceae*, and *Ruminococcaceae* [5].

Currently, findings regarding gut microbiota from different studies can be highly variable, which makes clinical diagnosis and treatment challenging. In addition, emerging evidence has reported significant differences in gut microbiota composition between faeces and mucosa because components from faeces do not necessarily reflect the direct interaction with mucosa [6]. Therefore, the gut microbiota composition obtained from mucosal-associated and faecal samples can be two different microbial niches [7]. Furthermore, faecal samples cannot provide reliable results on the metagenomic functions and composition of the mucosa-associated microbiota found on the multiple sites of the gut [8]. Since faecal samples have their own biostructure, faecal samples should be homogenised to ensure that a more representative, uniform sample can be obtained for analysis because of the heterogenous bacterial distribution within the faecal samples [9]. Moreover, due to different levels of pH, oxygen, antimicrobial compounds, and bile acid in the gastrointestinal (GI) tract, some of the dominant bacterial phyla including *Actinobacteria*, *Bacteroidetes*, and *Firmicutes* are not uniformly distributed throughout the GI tract [10]. A study by Wu et al. reported that 35% of the low-abundance taxa were detected only in one replicate but not in the second replicate [11]. The use of homogenised faecal samples can be used to reduce the intraindividual variation of faecal microbiota [12,13].

In the majority of cases, it is often unrealistic to analyse fresh faecal samples immediately and the storage of faecal samples needs to be considered. The practice of storing faecal samples at −80 °C without preservative is considered the gold standard used for profiling gut microbiota. This is because this practice can retain a gut microbiota composition similar to those of fresh faecal samples and avoids the adverse impacts of preservatives used [4]. However, if the storage temperature of −80 °C is unavailable due to certain logistical issues, the faecal samples can be transported and stored at 4 °C, which can minimise changes to the faecal microbiota’s composition [14]. There are also other storage methods that can be used with and without preservatives, which depend on the objectives and conditions of the studies. No differences in faecal microbiota profiles were observed when the faecal samples were stored at the following conditions: room temperature for 24 h; in Eppendorf tubes at room temperature for 3 days; and −20 °C for 7 days [4]. Faecal samples can also be stored using preservatives such as RNAlater and 95% ethanol [15]. With the proper understanding of sampling methods, we can delve into the composition and functionality of the gut microbiota.

## 3. Composition of Gut Microbiota

A decade ago, our knowledge of gut microbiota mostly stemmed from culture-based approaches. Conventional culture-based techniques have been used to isolate, identify, and enumerate the gut microbiota’s composition. However, these techniques are only able to isolate 10–25% of the microbiota because the majority of gut microorganisms are anaerobic. Anaerobic culturing techniques are therefore used for gut microbiota analysis, but these techniques are time-consuming [16].

More recently, the use of culture-independent methods including high-throughput, low-cost sequencing methods has significantly improved our understanding of the gut microbiota. The study of gut microbiota consists of 16S ribosomal RNA (rRNA)-based sequencing of bacterial genes and bioinformatics analysis. Since the bacterial 16S rRNA is present in all bacteria, the 16S rRNA gene is targeted to distinguish different species [17]. Metabolomics is another rapidly growing area of gut microbiota research, studying small molecules related to the interaction between host and bacterial metabolism that can reflect implications for health and disease states. [18]. The estimated number of microorganisms in the GI tract has been reported to be more than 10^14^ [19]. However, the ratio of human:bacterial cells has been estimated to be ~1:1 based on a recent estimation [20].

The gut microbiota has ≥1000 bacterial species; of these, *Bacteroidetes*, *Actinobacteria*, *Firmicutes*, and *Proteobacteria* are the dominant phyla (Table 2). *Bacteroidetes* and *Firmicutes* make up 90% of the gut microbiota, which is most abundant in a healthy gut. The genera of *Bacteroidetes* include *Prevotella* and Bacteroides. The *Firmicutes* phylum consists of ≥200 genera including *Bacillus*, *Lactobacillus*, *Ruminicoccus*, Enterococcus, and Clostridium. The less abundant taxa include *Bifidobacterium*, *Akkermansia*, and *Escherichia* [21]. However, there are strong inter-individual variations in the composition of the gut microbiota. In addition, the abundance of both common and rare taxa does not reflect its functional importance because the variations in taxa are associated with host function and health. It is important to note that due to high inter-individual variation, the study methodologies used, and participants recruited, the attempt to accurately identify and explain the gut microbiota composition in healthy individuals is challenging. Furthermore, the measured gut microbiota composition is isolated from faecal samples, which do not truly reflect the overall gut microbiota diversity of the GI tract. This is because the gut microbiota exhibits uneven distribution across the GI tract, with its composition varying by location. Consequently, faecal microbiota primarily represent the luminal microbiota of the colon, providing limited insight into the microbial communities of the small intestine. Faecal microbiota and mucosal-associated microbiota have been reported to represent two distinct microbial populations with differing compositions [19].

More advanced technologies have been developed for bridging the gap between the true gut microbiota diversity of the GI tract and faecal sample analysis. Smart capsules with sensing technologies have been used to address the limitations of current sampling methods [19]. These smart capsules offer minimally invasive access to previously inaccessible regions of the gut, made possible through the development of swallowable devices. With the use of the smart capsules, gut parameters such as pH, pressure, temperature, and gases at different segments of the gut can be measured to provide a more accurate assessment of gut performance. However, the expense of current smart capsule technologies also limits their accessibility for personal use, as they rely heavily on hospital infrastructure and specialised medical support [19]. Also, the size limitations of swallowable capsules currently restrict most sensing devices to measuring only a few gut parameters. As a result, smart capsules are typically designed for single, specialised tasks, limiting their ability to provide comprehensive data. Also, separate capsules are required to sense individual parameters, significantly increasing overall costs and placing a burden on healthcare systems [19]. Therefore, overcoming these constraints requires significant advancements in miniaturisation, enabling the integration of multiple sensing capabilities into a single capsule for holistic gut assessment.

Using data obtained from the European Metagenomics of the Human Intestinal Tract (MetaHIT) and the US Human Microbiome Project (HMP), 2172 species have been isolated from human beings and are categorised into 12 phyla; of these, 93.5% are classified as *Firmicutes*, *Actinobacteria*, *Proteobacteria*, and *Bacteroidetes* [22,23]. There were 386 identified species in humans that are anaerobic and found in mucosal regions (e.g., the GI tract and oral cavity) [22]. In the small intestine, facultative anaerobes such as *Proteobacteria* (*Enterobacteriales*) and *Firmicutes* (*Lactobacillales*) are commonly found [24]. Since the proximal small intestine has harsher environmental conditions, it has a lower microbial density. In addition, due to high concentrations of oxygen, acids, and antimicrobials along with short gastrointestinal transit time, the growth of microorganisms is limited in the small intestine [25]. In the large intestine, due to the slow gastrointestinal transit, this leads to increased availability of nutrient concentration (undigested complex carbohydrates from the small intestine), which support the growth of fermentative microorganisms [26]. Therefore, the highest microbial density is reported in the large intestine, which is predominantly colonised by the *Firmicutes* (*Ruminococcaceae* and *Lachnospiraceae*) and *Bacteroidetes* (*Rikenellaceae*, *Bacteroidaceae*, and *Prevotellaceae*) species [25]. It is suggested that the microbial density within the GI tract has limited the ability of pathogenic microorganisms to colonise the GI tract [10].

## 4. Functions of the Gut Microbiota

The gut microbiota has been known as an accompanying commensal to a ‘metabolic organ’, which can influence the functions in systemic inflammation, immunity regulation, and nutrition [27]. Table 3 shows some key functions of the gut microbiota with examples. There has been significant interest in gut microbiota in recent years, especially the relationship between the gut microbiota and a large array of diseases including obesity, IBS, and allergic diseases. It has been speculated that the gut microbiota plays significant functional roles in maintaining the normal functions of the gut. Several studies including European Metagenomics of the Human Intestinal Tract (MetaHIT) and the US Human Microbiome Project (HMP) have provided evidence to support the beneficial functions of the normal gut microbiota [28]. *Bifidobacterium infantis* (*B. infantis*) has a human milk oligosaccharide (HMO)-related gene cluster that is involved in the digestion of HMO [29].

### 4.1. Nutrient Metabolism

The gut microbiota processes dietary carbohydrates, producing short-chain fatty acids (SCFAs) vital for gut health. About 20% of the digested carbohydrates are resistant to amylase digestion. Fermentation of these indigestible carbohydrates, including non-starch polysaccharides and cellulose, by gut microbiota such as *Bacteroides*, *Bifidobacterium*, *Enterobacteria*, *Roseburia*, and *Fecalibacterium* results in the production of SCFAs including acetate, butyrate, and propionate [30]. These SCFAs are absorbed in the large intestine. They play several important roles in increasing the proliferation rate of epithelial cells and promoting tight junction integrity in the large intestine. Vitamin K is produced by gut microbiota, mostly in the ileum and absorbed from the gut. Vitamin B12 synthesised by gut microbiota is bound to R factor in the stomach, transferred to intrinsic factor in the small intestine, and absorbed in the terminal ileum [31]. The gut microbiota is also involved in breakdown of phenolic compounds. These compounds are glycosylated derivatives bounded with sugars such as galactose, glucose, ribulose, and arabinopyrinose, which remain inactive until the removal of the sugar moiety by gut microbiota to active compounds [32].

### 4.2. Immune Homeostasis

The gut microbiota, in interaction with the innate and adaptive immune systems, contributes to gut immunomodulation [33]. The two-tiered mucus layer in the large intestine is constituted by mucin glycoproteins, which are secreted by the intestinal goblet cells. The resistin-like molecule (RELM)-β and trefoil-factor are also produced by the intestinal goblet cells to maintain barrier integrity [34]. In the small intestine, the gut microbiota via its metabolites induces synthesis of antimicrobial peptides (AMP) such as C-type lectins and cathelicidins by Paneth cells through pattern recognition receptor (PRR)-mediated mechanisms. PRR are then activated by organism-specific microbe-associate molecular patterns (MAMP), which lead to the activation of signalling pathways that are involved in the synthesis of AMP and promotion of mucosal barrier function [10]. Local immunoglobulins are also induced by gut microbiota to prevent the overgrowth of pathogenic bacteria. Therefore, disrupted microbial homeostasis has been associated with several metabolic diseases and diseases including IBS, because normal immunological functionality can be compromised by microbial dysbiosis [35]. The gut microbiota serves as an important regulator of mucosal barrier repair in the intestines [10,36].

### 4.3. Other Functions

The gut microbiota imparts specific functions in xenobiotic and drug metabolism. A gut microbial metabolite p-cresol, which is produced from tyrosine in reactions involving gut microbiota, has been demonstrated to significantly reduce the liver’s capacity to metabolise acetaminophen. Therefore, it is imperative to explore which gut bacteria might have the potential to influence drug-induced responses and disease development, with the gut bacteria as the principal target of drug action [37]. The gut microbiota also contributes to the maintenance of the function and structure of the GI tract. The expression of the small proline-rich protein 2A induced by *Bacteroides thetaiotaomicron* is needed for maintaining desmosomes at the epithelial villi [38]. In addition, the gut microbiota induces transcription factor angiogenin-3 for the structural development of the gut mucosa [39].

## 5. Factors That Can Influence Gut Microbiota

**Figure 2:**
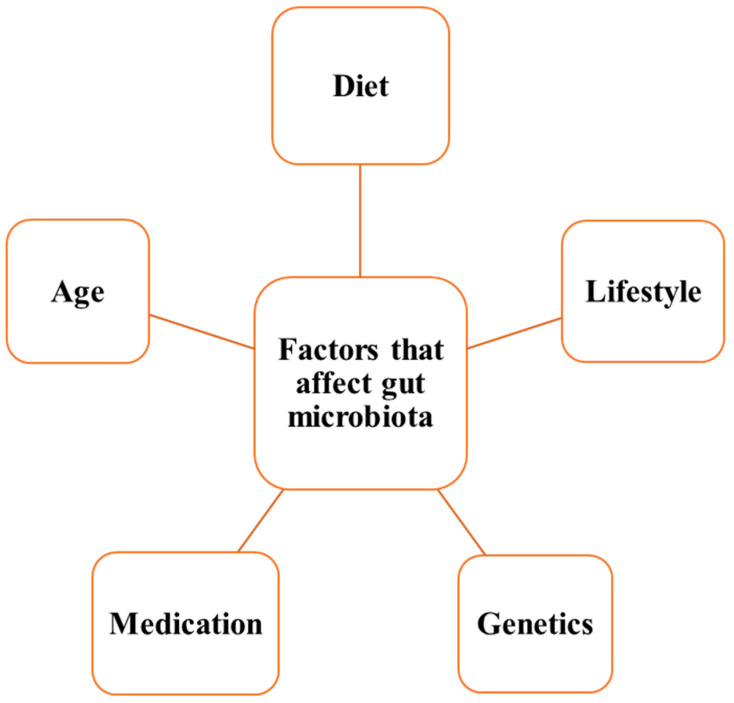
Factors that affect gut microbiota.

The composition of the gut microbiota can be affected by several factors, namely nutritional, immunological, and chemical gradients along the GI tract. In addition, other factors such as the mode of delivery (caesarean or vaginal), infant feeding, use of medication (in particular antibiotics), and lifestyle play a role in shaping the gut microbiota. According to the findings of a study including 1126 twins, genetics plays only a limited role in shaping gut microbiota composition (with a mean of 8.8%), suggesting that gut microbiota composition can be shaped throughout the life span [40] (Figure 2).

The incidence of infectious diseases has decreased over the last half century because of improved population health through immunisation, better healthcare services, and the implementation of water, sanitation, and hygiene (WASH) programmes [41]. However, at the same time, there has been a paradigm shift in the burden of diseases towards immune-mediated diseases such as allergy due to the significant changes in diet as part of the Western lifestyle [42]. The ‘hygiene hypothesis’ has suggested that altered exposure to microbial antigens in early childhood due to improved public health interventions could change the way individuals immunologically adapt to the external environment [43,44]. As a result, this has led to increased risks of inflammatory disease development and immune system dysfunction.

During pregnancy, bacteria from the maternal gut can pass through the placenta from the mother’s bloodstream into the amniotic fluid [45]. This has been evidenced by the identification of maternal gut microorganisms in both umbilical cord blood and meconium [46,47]. Upon birth, full-term newborns delivered vaginally would be exposed to maternal vaginal and colonic bacteria [48]. On the other hand, if the newborns are delivered by caesarean section, their gut microbiota lacks presence and diversity within the *Bacteroidetes* phylum. This might be due to the colonisation of the infant gut by environmental microorganisms rather gut microorganisms. Newborns delivered by caesarean section have a higher risk of developing obesity and asthma, probably due to the gut microbiota [49]. The GI tract needs to be colonised before the development of an adequate immune function in order to develop a balanced innate and adaptive immune system [50]. In infants, the gut microbiota composition changes dramatically shortly after birth and during breastfeeding, followed by a second shift when solid foods are introduced. Until 2–3 years of age, infants’ gut microbiota composition is exposed to low microbial diversity but with a high rate of microbial change. Therefore, this time period is critical for the development of gut microbiota in infants because it plays a fundamental role in host health later in life, which is associated with disturbances linked to increased risk of metabolic disorders in adulthood. Although the gut microbiota composition can still be influenced after this window, the resilience of the gut microbiota to perturbations is dependent on the responsiveness of its intrinsic core taxa to return to its original state of equilibrium [51].

Long-term use of a large number of broad-spectrum antibiotics may cause the gut microbiota to lose the diversity and resilience that are required for establishing a balanced immune response. This could potentially lead to the development of inflammatory and autoimmune disorders when the symbiotic relationship between the gut microbiota and the host is disrupted. In addition, a major concern related to the overuse of antibiotics is that antibiotic-resistant genes could be horizontally transferred and lead to reservoirs of multidrug-resistant gene pools in organisms [52].

## 6. Impact of Diet on Gut Microbiota

Diet is one of the most extensively studied factors and is thought to account for ≥20% of microbial structural variations, indicating the potential of dietary interventions in treating diseases by modulating gut microbiota. In addition, dietary patterns are reported to correspond with the microbial composition. Since gut microorganisms are purged abundantly and can double their numbers within an hour, gut microbiota composition is thought to change rapidly at the species and family level within 24–48 h following the delivery of dietary interventions [53]. Similar observations have been reported in studies using mouse models that demonstrated the alterations of gut microbiota within a day after manipulating the macronutrient intake [54,55,56]. Although short-term, drastic dietary interventions have been reported to rapidly alter gut microbiota diversity, these changes were temporary and only persisted for a short period. Therefore, a long-term, habitual dietary intake may play an important role in shaping gut microbiota [54]. Habitual dietary patterns rich in legumes, bread, fish, and nuts are associated with a reduced abundance of opportunistic bacterial clusters and decreased activity of pathways involved in endotoxin synthesis and stool inflammatory markers. Higher abundances of beneficial commensals such as *Roseburia*, *Faecalibacterium*, and *Eubacterium* spp. are associated with the consumption of key components of the Mediterranean diet, including nuts, oily fish, fruits, vegetables, cereals, and red wine, across all cohorts. These bacteria are known for their anti-inflammatory effects in the intestine through the fermentation of dietary fibre into short-chain fatty acids. In addition, enterotypes have been strongly linked to long-term dietary patterns, with diets high in protein and animal fat associated with *Bacteroides* dominance, while carbohydrate-rich diets favoured *Prevotella* [56].

However, limited evidence is available on the duration of period needed for dietary interventions to enable permanent alterations to the ecological homeostasis of gut microbiota [57]. This would then lead to a new state of ecological homeostasis by causing other species to proliferate and inducing new species, which can be beneficial to the host health. In addition, the diversity of gut microbiota and the richness of beneficial taxa can be improved. The interactions between diet and gut microbiota need to be taken into consideration to ensure the continuous availability of the substrate needed by the gut microbiota [58].

A review of the literature has demonstrated that changes made in diet can have a significant impact on the gut microbiota, which is mainly influenced by the types, amounts, and contents of dietary fibres from fruit and vegetables [59,60]. Fructans and galactooligosaccharides (GOS) have been reported to increase the abundance of *Lactobacillus* and *Bifidobacterium* species in faecal samples [60]. The effects of other dietary components such as protein on the gut microbiota have also been extensively investigated and reported in the literature. The consumption of protein has been associated with overall gut microbial diversity. For example, the consumption of whey decreased the pathogenic *Bacteroides fragilis* and *Clostridium perfringens*, while whey and pea protein extract increased gut-commensal *Bifidobacterium* and *Lactobacillus*. In addition, dietary polyphenol intake can influence gut microbiota composition and microbial metabolite profiles by modulating the bacterial 7α-dehydroxylation process, which converts deconjugated primary bile acids (BAs) into secondary BAs, thereby altering the secondary BA composition [61,62].

The Western diet, characterised by high fat and low fibre intake, is associated with decreased microbial diversity and richness, which are key indicators of a healthy gut microbiota. This dietary pattern is linked to a reduction in *Bacteroidetes* and an increase in *Firmicutes* and *Proteobacteria*, microbial shifts that contribute to obesity, diabetes, chronic inflammation, and a range of chronic diseases including cardiovascular dysfunction, systemic metabolic disorders, and digestive tract conditions [61]. High-fat diets specifically increase the abundance of *Alistipes* and *Bacteroides* species while reducing beneficial commensals such as *Faecalibacterium* (a key producer of butyrate). In contrast, omega-3 polyunsaturated fatty acids (PUFAs) support the growth of butyrate-producing bacteria and promote *Bifidobacterium* and *Lactobacillus*, both of which are associated with anti-inflammatory effects. Similarly, long-term consumption of animal-protein-rich diets has been linked to an increased abundance of *Alistipes* and *Bacteroides* but a reduction in *Roseburia* (another butyrate producer essential for gut health) [61]. Conversely, plant-based protein sources promote the growth of *Lactobacillus* and *Bifidobacterium* and are associated with higher alpha diversity and enrichment of beneficial taxa such as *Bacteroidetes*, *Prevotella*, and *Roseburia* [61,62].

Evidence is accumulating that the gut microbiota composition is primarily dependent on long-term dietary patterns. However, many published studies have been cross-sectional in design, often relying on food frequency questionnaires (FFQ) to assess habitual dietary patterns [58]. Habitual dietary patterns are usually established in adulthood, when there is less inclination to try new foods [63]. However, the quantity and quality of nutrients consumed in adults can still influence gut microbiota composition. This is because the habitual dietary patterns supply gut microbiota with a constant food source and influence the gut’s microbial ecology, as evidenced by the relationship between habitual dietary patterns and specific enterotype composition [55].

There is increasing interest in the composition of breast milk and its influence on gut microbiota composition. One of the important reasons for this is the billion-dollar infant formula market [64]. In breast-fed children, the dominant intestinal microbiota includes *Bacteroide*, *Lactobacilli*, and *Bifidobacteria* and has lower microbial diversity when compared with infant formula-fed children [65]. As prebiotics, HMOs are not digested by pancreatic enzyme and are identified as the third most abundant dietary component of breast milk [66]. HMOs promote the growth of *Bifidobacteria*, *Bacteroides*, and *Lactobacillus* in the large intestine [67]. In addition, SCFA produced from the digestion of HMOs are used as an energy source and decrease the luminal pH, which inhibits colonisation by pathogens [68].

## 7. Gut Microbiota and Obesity

Obesity is one of biggest current public health problems, with its prevalence increasing rapidly on a global scale. Obesity has been associated with increased risks of cardiovascular disease (CVD), type 2 diabetes, and mortality risk. The relationship between the gut microbiota and obesity was initially suggested based on findings from studies in germ-free mice. The conventionally reared mice had higher levels of body fat and gonadal fat than germ-free mice [69]. Obese infants were reported to have fewer *Bifidobacteria* and higher prevalence of *Staphylococci* in their first year of life. It is possible that excessive weight gain may start to happen in the foetal period because overweight mothers provide excessive energy to the foetus. In addition, a vicious cycle of adverse metabolic development may occur when the altered gut microbiota composition of an obese pregnant woman is transferred to the foetus. Specific shifts in gut microbiota composition have been reported with maternal body mass index (BMI) and weight gain. Infants born to women with normal weight gain during pregnancy have higher levels of *Bifidobacterium* than those born to women with excessive weight gain, indicating that there is an association between the gut microbiota and maternal nutritional status during pregnancy.

Emerging evidence suggests that the gut microbiota is intrinsically associated with overall health outcomes, including the risk of obesity and obesity-related metabolic disorders [70]. In addition, since the gut microbiota is involved in regulating energy balance and metabolism, it is a crucial cause of obesity. The gut microbiota metabolises indigestible polysaccharides and complex carbohydrates to SCFAs, including acetate, butyrate, and propionate. Acetate and propionate are needed for gluconeogenesis and lipogenesis in the liver, while butyrate serves as the primary energy source for epithelial cells in the large intestine. For example, undigested dietary fibres such as arbinases and xylanases (primary substrates for producing SCFA) are broken down into monosaccharides (primarily pentoses and hexoses) through the action of glycoside hydrolases [69]. The resulting monosaccharides are metabolised by the gut microbiota via the pentose phosphate pathway (for pentoses) or glycosis (for hexoses). SCFAs are absorbed by epithelial cells either passively or through active transport, primarily via the monocarboxylate transporter 1 (MCT-1) and, to a lesser extent, the sodium-coupled monocarboxylate transporter 1 (SMCT-1). Among SCFAs, butyrate is particularly significant, as it serves as the primary energy source for colonocytes, meeting 60–70% of their energy demands [69]. In addition, the gut microbiota releases metabolites which play a role in controlling appetite through directly affecting the central nervous system or indirectly via modifying hormone secretion [50]. In obese individuals, the production of glucagon-like peptide-1 (GLP-1) which is responsible for the uptake of energy in the GI tract, is reduced, leading to the development of insulin resistance. Obese individuals were reported to have a higher *Firmicutes*/*Bacteroidetes* ratio (F/B); higher *Fusobacteria*, *Firmicutes*, *Lactobacillus* (*reuteri*), *Mollicutes*, and *Proteobacteria*; and fewer *Methanobrevibacter smithii*, *Verrucomicrobia* (*Akkermansia muciniphila*), *Bacteroidetes*, *Faecalibacterium* (*prausnitzii*), *paracasei*, and *Lactobacillus plantarum* than individuals with normal weight [71].

An elevated F/B ratio, particularly values exceeding 3.0 as reported in numerous studies, has been consistently associated with excess body weight. This increased F/B ratio is a hallmark of gut dysbiosis linked to obesity and related metabolic and inflammatory disorders, including type 2 diabetes, metabolic-associated fatty liver disease (MAFLD), gout, and dyslipidaemia [69]. The phylum Firmicutes is often regarded as more metabolically efficient due to its enhanced capacity to extract energy from dietary polysaccharides, which is associated with higher caloric intake and diets rich in protein, fat, and sugar. Conversely, the phylum Bacteroidetes is less metabolically efficient and is linked to diets high in dietary fibre [69]. Therefore, this microbial imbalance contributes to greater energy harvesting, systemic inflammation, and metabolic dysregulation, which are central to the pathophysiology of obesity.

## 8. Future Perspectives

Future microbial research should focus on the roles and functions of the gut microbiota in early features of brain development and behaviours. These include neurobehaviours in the pathophysiology of autism spectrum disorder [72]. In addition, we should try to decode the signals and language by which gut microbiota and the host communicate in health. This is because a better understanding of the crosstalk could lead us to better knowledge of the development of some common multifactorial diseases which are related to the gut dysbiosis [73].

The application of metabolomics, metagenomics, and metaproteomics has provided a better characterisation and quantification of the genomes of the microorganisms and the potentially bioactive metabolites they modify or secrete. The identification of these bioactive metabolites may be used to highlight how dietary pattern affects specific disease progression [74]. The vision for the future in gut microbiota research is more likely to be ‘omics’-based, because these approaches will provide further insight into interactions between gut microbiota and the host. In addition, some of these bioactive metabolites can cross the blood–brain barrier and might be potential metabolic signals used for communication between the gut microbiota and the host or between the microorganisms [75]. With the use of machine learning and artificial intelligence (AI), the genomic data and thousands of bioactive molecules produced by the gut microbiota can be analysed to study the potential drivers of gut-related diseases. A random forest classifier utilising log-transformed operational taxonomic unit (OTU) data from oral and faecal microbiota has been developed to differentiate individuals with colorectal cancer from healthy controls [76]. Furthermore, this can be used to investigate how gut microbiota can influence the response to disease treatment and overall host immunity in patients diagnosed with cancer. This includes, for example, metabolite profiling of patients who undergo faecal transplant and immunotherapy. The roles of the tumour microbiome should also be explored because of the roles of bacteria and viruses in carcinogenesis and in the response to the treatment therapy [77]. Also, personalised nutrition has been reported to modulate gut microbiota, thereby improving overall health outcomes. Individual variability in gut microbiota composition and function underpins differences in dietary responsiveness, positioning microbiota-driven approaches as a promising avenue for advancing personalised nutrition and health management strategies [78].

Although there is a broad understanding of gut microbiota and there have been plenty of studies that have reported the relationship between gut microbiota and certain health outcomes, these studies were mainly correlation studies, which did not prove causation. Therefore, there is an urgent need to move from correlation to causation when exploring whether gut microbiota composition is the cause of the disease. The understanding of gut microbiota is essential for the development of personalised diagnostic, medicinal, and nutritional strategies.

## 9. Conclusions

Our review underscores the critical role of the gut microbiota in health and disease with a focus on obesity, highlighting its influence on metabolic regulation, inflammation, and host–microbiota interactions. Our key findings reveal that dietary patterns significantly shape microbial diversity and functionality, with implications for managing conditions such as obesity. Emerging technologies, including multi-omics and AI-driven approaches, offer unprecedented insights into the microbiota’s composition and function, enabling personalised dietary interventions and microbiome-based therapies. On the other hand, our review demonstrates that there is still a large knowledge gap in regard to the role of gut microbiota in nutrition and health. The gut microbiota benefits the host by supporting nutrient metabolism, regulating immunity, and protecting against pathogens. These functions and mechanisms can be disrupted due to an altered gut microbiota composition (dysbiosis). Other microbiomes such as the respiratory system microbiome, urogenital microbiome, and skin microbiome should also be extensively studied because they are equally important in providing critical functions to the relevant organ. The modulation of these microbiomes by therapeutic agents in cancer treatment would open up a promising new treatment strategy, particularly in the context of gut-related diseases.

## Figures and Tables

**Table 1:** Sampling methods for gut microbiota.

Types of Sampling Methods	Advantages	Drawbacks	Use Cases
Biopsy	Precise description of gut microbiota; sampling site needed	Invasive, expensive; bowel preparation	Disease pathology and histopathological analysis
Faeces	Non-invasive; convenient; inexpensive	Uneven distribution of microorganisms within the faeces samples	Non-invasive diagnostics and population-level studies
Luminal brush	Precise description of gut microbiota; sampling site needed	Invasive, expensive; bowel preparation	Localised sampling and biofilm analysis
Surgery	Precise description of gut microbiota; sampling site needed	Preoperative preparation needed	Severe disease research and histological studies

**Table 2:** Examples of the taxonomic composition of the gut microbiota.

Phylum	Class	Order	Family	Genus	Species
*Actinobacteria*	*Actinobacteria*	*Bifidobacteriales*	*Bifidobacteriaceae*	*Bifidobacterium*	*Bifidobacterium longum*
*Bacteroidetes*	*Bacteroidia*	*Bacteroidales*	*Bacteroidaceae*	*Bacteroides*	*Bacteroides fragilis*
					*Bacteroides uniformis*
			*Prevotellaceae*	*Prevotella*	*Prevotella* spp.
			*Rikenellaceae*	*Alistipes*	*Alistipes finegoldii*
			*Tannerellaceae*	*Parabacteroides*	*Parabacteroides distasonis*
*Firmicutes*	*Bacilli*	*Lactobacillales*	*Lactobacillaceae*	*Lactobacillus*	*Lactobacillus reuteri*
		*Bacillales*	*Staphylococcaceae*	*Staphylococcus*	*Staphylococcus leei*
	*Clostridia*	*Clostridiales*	*Clostridiaceae*	*Clostridium*	*Clostridium* spp.
*Fusobacteria*	*Fusobacteriia*	*Fusobacteriales*	*Fusobacteraceae*	*Fusobacterium*	*Fusobacterium nucleatum*
*Proteobacteria*	*Gamma proteobacteria*	*Enterobacterales*	*Enterobacteriaceae*	*Shigella*	*Shigella flexneri*
				*Escherichia*	*Escherichia coli*
*Verrucomicrobia*	*Verrucomicrobiae*	*Verrucomicrobiales*	*Akkermansiaceae*	*Akkermasia*	*Akkermansia muciniphila*

**Table 3:** Some key functions of gut microbiota with examples.

Functions	Descriptions	Examples
Metabolic functions	Breakdown of complex carbohydrates and dietary fibres into SCFAs.	Production of acetate, propionate, and butyrate for energy and metabolic regulation.
Nutrient synthesis	Biosynthesis of essential nutrients and vitamins.	Synthesis of vitamin K and B vitamins (e.g., B12).
Immune system modulation	Regulation of immune responses and maintenance of immune tolerance.	Interaction with regulatory T cells to reduce inflammation; SCFAs promote anti-inflammatory pathways.
Pathogen defence	Competitive exclusion of pathogens and production of antimicrobial compounds.	Production of bacteriocins; inhibition of *Clostridioides difficile*.

## Data Availability

No new data were created or analyzed in this study.
